# Improved Speech Authenticity Detection in Chinese–English Bilingual Contexts

**DOI:** 10.3390/s24216807

**Published:** 2024-10-23

**Authors:** Cheng-Yuan Tsai, Sheng-Chain Chang, Chao-Hsiang Hung, Syu-Siang Wang, Shih-Hau Fang

**Affiliations:** 1 Forensic Science Division, Ministry of Justice Investigation Bureau, New Taipei City 231, Taiwan; m55050@mjib.gov.tw; 2 Department of Electrical Engineering and AI Research Center, Yuan-Ze University, Taoyuan City 320, Taiwan; s1104633@mail.yzu.edu.tw (S.-C.C.); s1108501@mail.yzu.edu.tw (C.-H.H.); 3 Department of Electrical Engineering, National Taiwan Normal University, Taipei City 106, Taiwan

**Keywords:** acoustic signal processing, machine learning, forgery detection, audio tampering, signal analysis, deep learning

## Abstract

The rapid evolution of voice technology has heightened the need for robust detection systems to distinguish between authentic and tampered speech. Recent competitions have significantly advanced the development of countermeasures against spoofing attacks. However, while advancements in detection technologies have been notable, existing methods often focus on a single type of tampering and language. Our contribution lies in developing an improved model that integrates an enhanced ResNet architecture with an LSTM to improve the detection of tampered audio, particularly in challenging multilingual scenarios. In the experiments, we built a hybrid dataset from self-recording Chinese speech and public VCTK2 English samples, enhanced the ResNet model generalization capabilities, and evaluated our approach using the bilingual dataset. Experiment results demonstrate that the proposed approach achieves a superior performance with an equal error rate of 11.62%, even in the face of bilingual conditions, and, more importantly, outperforms the leading models from ASVSpoof 2021 and ADD 2022 competitions. We also employed advanced tampering techniques, including CycleGAN voice conversion and auto splicing, to simulate real-world tampering scenarios and verify the effectiveness of the proposed approach.

## 1. Introduction

In recent years, the rapid advancement of voice technology has brought authentic versus tampered speech detection to the forefront of research [1]. The methods for manipulating audio files have progressed from simple automatic clipping to more sophisticated techniques like speaker conversion and generation [2]. Although detection technologies have made significant strides, many current methods remain focused on identifying only one type of tampering [3].

Competitions, such as ASVSpoof 2021 [4], have played a crucial role in advancing the study of spoofing and the development of countermeasures to protect automatic speaker verification systems from manipulation. ASVSpoof 2021 was the fourth in a series of bi-annual challenges that addressed these issues. The challenge employed two primary techniques for manipulating speech: text-to-speech (TTS) and voice conversion (VC). While TTS and VC technologies have been developed to benefit industries such as multimedia and robotics, they have also been exploited for malicious purposes, including generating fake news, identity impersonation, and telephone scams. This has underscored the importance of synthetic voice detection as a critical research area within spoofing countermeasures.

The ADD 2022 [5] competition further supported the advancement of anti-spoofing systems by offering more realistic data and various challenging scenarios. For instance, in low-quality fake audio detection, data comprises a mix of real and spoof-attack-generated VC and TTS audio. These tasks represent more realistic scenarios researchers must navigate in their ongoing efforts to improve detection technologies.

In our increasingly globalized and diverse world, it is common to encounter regions with multiple languages [6]. Therefore, the ability to detect the authenticity of speech should not be hindered by language differences. Cross-language verification has become a pressing issue that needs addressing to ensure robust and reliable speech verification systems [7]. However, this problem presents several challenges [8]. Speech verification must consider various aspects, including tampering methods such as automatic clipping, speaker conversion, and the mixture of languages like Mandarin and English. Each of these scenarios requires different handling and robust detection techniques [9].

Previous research has primarily utilized ResNet [10] models for speech verification. While ResNet is a popular and effective model structure, it has shown performance disparities when dealing with different tampering methods and mixed-language scenarios. This indicates a need for further research to improve the model’s generalization and robustness. The main drawback of previous approaches is that the ResNet model is typically limited to handling a single language effectively. To address this limitation, we propose an enhanced approach that combines an improved ResNet model with an LSTM [11] to process time series data. This integration aims to improve the model’s performance in detecting tampered audio files, especially in cases involving mixed Mandarin and English speech datasets [12].

We collected bilingual datasets in Chinese and English to facilitate our analysis. The Chinese dataset consists of 320 sentences that are commonly used daily, selected from the Taiwan Mandarin Speech Hearing in Noise Test (TMHINT) [13]. Each sentence comprises ten characters, covering most pronunciation types commonly encountered in everyday life. The recording process involved 24 speakers, comprising 19 men and 5 women. This dataset employs two primary tampering techniques: CycleGAN voice conversion, which modifies voice characteristics to simulate different speakers [14,15,16], and auto splicing, which automatically combines audio segments to create tampered samples. Both tampered and original data are stored in a database for subsequent analysis. For the English dataset, we utilized the publicly available VCTK2 dataset [17], selecting five male and five female speakers, and generated a total of 6400 English sentences.

In the proposed detection pipeline, Linear Frequency Cepstral Coefficients (LFCCs) are initially extracted from the input speech data as feature representations. These features are subsequently passed through a ResNet architecture, comprising multiple residual blocks, followed by further analysis with a Long Short-Term Memory (LSTM) network, enabling the distinction between authentic and manipulated speech signals. Our contributions include enhancements to the ResNet model, particularly through the exploration of temporal characteristics, which collectively improve the model’s robustness and generalization across diverse languages and datasets [18]. Specifically, we modified the ResNet structure by eliminating the second fully connected layer during the testing phase, a step that significantly boosted the model’s generalization capabilities. Experimental evaluations reveal that the proposed model achieves an Equal Error Rate (EER) of 11.62%. Moreover, when applied to the simultaneous detection of two languages, our method outperforms the first-place winners in the ASVSpoof 2021 and ADD 2022 competitions. These results underscore the efficacy of our novel framework in the domain of tampered speech detection.

Despite these advancements, our method has certain limitations. Future directions include integrating multimodal features (audio and text) to enhance speech detection accuracy and expanding the scope to include more languages beyond Chinese and English, thereby increasing the model’s versatility and robustness [19]. By addressing these aspects, we aim to develop more effective and versatile speech verification systems capable of handling the complexities of modern multilingual and multi-method tampered audio environments [20].

## 2. Related Work

Current research on voice authenticity detection predominantly focuses on developing technologies to identify various voice tampering methods. As technology advances, these methods have evolved from basic clipping to more complex techniques such as speaker switching and speech generation. Despite significant progress, most detection techniques still primarily focus on a single type of tampering. Numerous competitions related to authenticity detection have been organized. In this section, we will discuss two prominent voice authenticity detection competitions and explore the innovative approaches employed in these events.

### 2.1. Voice Authentication Competitions

ASVSpoof 2021 and ADD 2022 were two significant voice authentication competitions aimed at advancing the development of voice security technologies, particularly in response to the challenges posed by the continuous advancement of synthesis technologies. These competitions provided a valuable platform for participants to share innovative solutions and successful practices, thereby enhancing the applicability of voice authentication technologies in real-world scenarios.

The ASVSpoof 2021 competition highlighted the effectiveness of deep learning models, such as ResNet, in detecting automatically edited audio files. The first-place winner of ASVSpoof 2021 achieved an EER of 15.64% using a ResNet model, which leveraged its powerful deep learning capabilities to capture intricate patterns and anomalies within audio data, thereby distinguishing between genuine and spoofed recordings with high accuracy [4,21,22,23].

Similarly, the ADD 2022 competition focused on the detection of fake audio files generated by speaker switching. The winning team also utilized a ResNet model, achieving an EER of 11.98% in a monolingual context. However, when the test was extended to a bilingual setting, the model’s performance declined significantly, underscoring the challenges faced by speech authentication techniques in multilingual contexts. This drop in performance emphasized the need for more robust models capable of generalizing across different languages and dialects [5,24,25]. The ADD 2022 competition further highlighted the complexities of handling diverse linguistic features and accents, which are critical for global applications of voice authentication systems. To address these challenges, researchers have explored various techniques, such as transfer learning and multi-task learning, aiming to develop systems that maintain high accuracy regardless of linguistic variations [26].

### 2.2. ResNet

ResNet, short for Residual Network, has been a cornerstone in the development of deep learning models for voice authentication. The primary innovation of ResNet is its incorporation of residual blocks, where inputs are added directly to the outputs of stacked layers via shortcut connections [27]. This architectural design allows the network to learn residual functions concerning the input, facilitating the training of very deep models by mitigating the vanishing gradient problem.

The ResNet architecture typically includes convolutional layers followed by batch normalization and ReLU activations, with the output then being added back to the input to form a shortcut. This structure not only preserves feature integrity and accelerates convergence but also enhances the network’s performance across various domains, such as image recognition, natural language processing, and speech recognition. Consequently, ResNet has become a highly versatile tool in deep learning, playing a crucial role in the advancement of voice authentication technologies.

## 3. The Proposed Method

### 3.1. Flow Chart of the Proposed Method

**Figure 1:**
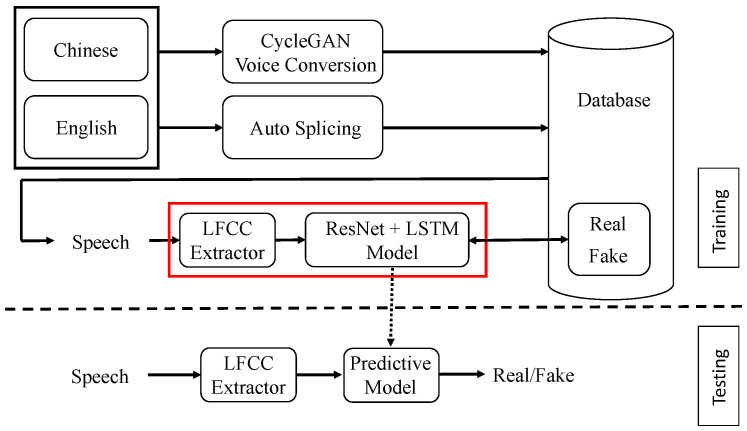
Application of ResNet in Chinese and English bilingual speech forgery detection block diagram.

**Figure 2:**
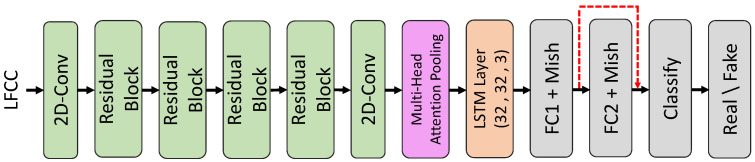
Details of our improved model.

In this study, we propose an enhanced approach for detecting speech forgery in bilingual datasets, specifically targeting Chinese and English. Our methodology integrates LFCC extraction with a combined framework of a ResNet and an LSTM model. The block diagrams presented in Figure 1 and Figure 2 illustrate the overall system and provide detailed architectural insights into the proposed approach.

As illustrated in Figure 1, the system processes speech data through several stages, accommodating speech inputs in both Chinese and English. Two primary tampering methods are employed to challenge the system’s detection capabilities. The first method, CycleGAN voice conversion [14,15,16], is used for speaker conversion, which modifies the voice characteristics of one speaker to mimic another. This method is particularly effective for generating fake speech that simulates different languages and inflections, making it ideal for testing the system’s robustness across linguistic variations. The second method involves auto splicing, which is executed through a Python program designed to automate the splicing process. This includes three types of edits. Delete: remove a specific part of the audio file. Same splicing: editing within the same audio file. Different splicing: editing different audio segments from the same person. Both tampered and original data are subsequently stored in a database for comprehensive analysis. This will be described in detail in Section 4.

In the detection pipeline, LFCC features are initially extracted from the speech data. These features are subsequently processed through the ResNet model, which comprises several residual blocks. The processed data are then analyzed by the LSTM model to differentiate between authentic and forged speech. Figure 2 provides a detailed view of the red-boxed section from Figure 1, emphasizing the LFCC feature extraction and the subsequent integration of the ResNet and LSTM models.

### 3.2. ResNet Model Improvement

Our decision to use ResNet was primarily inspired by the first-place solution from the ADD 2022 competition, which demonstrated ResNet’s effectiveness in voice authentication and tampered audio detection tasks. ResNet’s residual connections effectively address the vanishing gradient problem, making it well-suited for deep networks and complex tasks such as detecting tampered audio in cross-language scenarios. It has also proven robust in extracting high-level features from spectrograms.

While ResNet excels at capturing spatial features, we sought to enhance the model’s capacity to capture temporal dependencies in the audio data. To achieve this, we introduced an LSTM layer following the ResNet module. LSTMs are well-known for their ability to model sequential data and long-term dependencies, which are crucial in time series tasks like speech processing. By combining ResNet’s powerful feature extraction with LSTM’s temporal modeling capabilities, we aimed to improve tampered audio detection, particularly in multilingual and cross-language settings, where time-dependent patterns may vary significantly.

The architecture comprises several key components. First, LFCC features are extracted from the input speech data. These features are then processed through two-dimensional convolutional layers to capture spatial information. The four residual blocks further enhance feature extraction and provide the model with additional depth without encountering vanishing gradient issues. Multi-head attention pooling is applied to aggregate the features while emphasizing the most relevant parts of the input. Three LSTM layers (configured as 32, 32, 3) are used to process the sequential nature of the speech data, improving temporal feature extraction. Fully connected layers, utilizing Mish activation, refine the features, with Mish activation offering enhanced non-linear transformations. The final classification layer categorizes the processed features as either real or fake speech, completing the tampered audio detection process.

Continuing from the previous analysis, Figure 2 provides a detailed block diagram of the improved approach for detecting speech forgery in bilingual datasets, specifically targeting Chinese and English. This diagram delves into the inner workings of the red-boxed section from Figure 1, illustrating the key components and the flow of data through the system.

The process begins with the extraction of LFCC from the input speech data, crucial for capturing the essential characteristics of the audio signal. The LFCC is computed using the following Equation (1):

(1) $$LFCC\left(n\right)=\sum_{k=1}^{K}\left(\log|S\left(k\right)|\cdot \cos\left(\frac{\pi (n-0.5)k}{K}\right)\right),$$

where S(k) represents the magnitude spectrum of the speech signal, and *K* denotes the number of filter banks used in the feature extraction process. In this equation, S(k) is derived from the Fourier transform, capturing the magnitude at different frequency bins. *K* controls the resolution of the frequency analysis, determining the number of filters applied to the signal. The term log|S(k)| compresses the dynamic range of the spectrum, ensuring that variations in amplitude are more manageable for further processing. The cosine component is part of the discrete cosine transform (DCT), which decorates the frequency components and reduces redundancy. The result is a set of cepstral coefficients indexed by *n*, each representing different aspects of the frequency structure of the signal. These LFCC features are crucial for downstream tasks, often being fed into convolutional neural networks to capture spatial patterns in the data, which are essential for recognizing speech structures and patterns critical to tasks such as voice authentication or tampering detection.

Following this, multiple residual blocks, each comprising convolutional layers with skip connections, mitigate the vanishing gradient problem and enable the construction of deeper networks. These blocks enhance feature extraction by learning complex representations of the input data. After the residual blocks, the data are processed by a multi-head attention pooling layer, which aggregates features from different parts of the input while emphasizing the most relevant information. This layer enhances the model’s ability to focus on critical aspects of the speech signal.

The output from the attention pooling layer is subsequently passed through three LSTM layers configured as (32, 32, 3). These layers are adept at handling sequential data and capturing temporal dependencies, which are essential for analyzing speech data. The processed features are further refined through fully connected layers equipped with the Mish activation function, described by the following Equation (2):

(2) $$\mathrm{Mish}\left(x\right)=x\cdot \tanh\left(\mathrm{softplus}\left(x\right)\right)=x\cdot \tanh\left(\ln\left(1+e^{x}\right)\right)$$

where *x* is the input variable. The function combines the hyperbolic tangent (tanh) with the softplus function, which is a smooth approximation of the ReLU function, defined as $\ln(1+e^{x})$. This formulation helps ensure that the output grows logarithmically when *x* is positive. Mish has gained popularity in deep learning due to its smooth, non-monotonic nature, which allows it to capture complex patterns more effectively, while still maintaining stability during the backpropagation process in neural networks.

By integrating LFCC extraction, two-dimensional convolutional layers, residual blocks, multi-head attention pooling, LSTM layers, and fully connected layers with Mish activation, the proposed approach effectively captures both spatial and temporal features of the speech data. This comprehensive architecture ensures robust and accurate detection of speech forgery, adeptly addressing the complexities inherent in bilingual datasets and various tampering methods.

## 4. Experimental Setup

### 4.1. Chinese and English Datasets

The dataset used in this study encompasses both Chinese and English sentences. For the Chinese segment, the dataset includes 320 commonly used sentences selected from the TMHINT. Each sentence contains ten characters, representing a broad range of commonly used pronunciation types. The recordings involved 24 speakers, 19 males and 5 females, aged between 23 and 28 years, each tasked with reading sentences in sequence from the TMHINT. The recitations, with a fixed duration of 3 to 4 s per sentence, were recorded at a sampling rate of 48 kHz. These participants were selected for their lack of pronounced pronunciation difficulties or oral expression issues, thus reflecting the typical characteristics of the target speaker population. To maintain data balance given the smaller number of female speakers, only five male speakers were chosen, each contributing 320 sentences. Furthermore, an additional 3200 synthetic sentences were generated using the CycleGAN voice conversion model, resulting in a total of 6400 Chinese data samples.

For the English dataset, we utilized the publicly available VCTK2 dataset, selecting 5 male and 5 female speakers, which resulted in a total of 6400 English sentences. This selection process was carefully designed to match the number of Chinese samples, ensuring a balanced dataset for model training. The dataset was then randomly divided into three parts: 60% for training, 20% for validation, and 20% for testing. This random split allowed the model to be exposed to a diverse range of data during training, while also ensuring it was tested on unseen data to evaluate its generalization performance.

**Figure 3:**
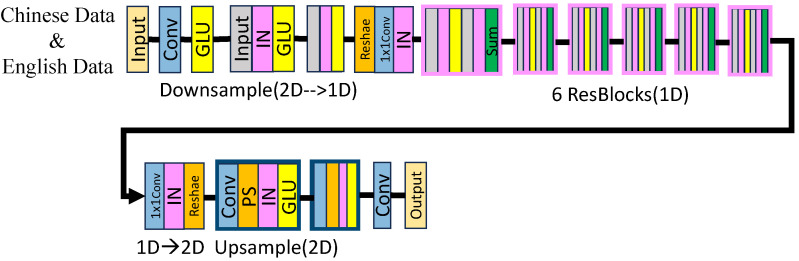
CycleGAN voice conversion model on Chinese and English datasets for voice authentication.

Figure 3 illustrates the CycleGAN voice conversion model used for generating synthetic Chinese sentences. This model comprises two generators (G and F) and two discriminators. The generators are equipped with convolutional layers utilizing the ReLU activation function, designed to transform the voice characteristics efficiently. Conversely, the discriminators feature convolutional layers with an increasing number of filters and employ the Leaky ReLU activation function to effectively differentiate between real and converted voices. The model was trained using the Adam optimizer, with an initial learning rate of 0.0002, throughout 100 epochs. The training process incorporated several loss functions, including cycle consistency loss, adversarial loss, and identity mapping loss, each contributing significantly to the creation of high-quality synthetic sentences.

There were three principal types of auto splicing techniques utilized in the manipulation of audio files, each applied to an original dataset consisting of 3200 sentences. Deletion (3200 sentences): this involves removing sounds from specific areas within the audio file. Application: this is typically used to eliminate noise, meaningless speech, or other unwanted information, enhancing the clarity of the audio. Different splicing (3200 sentences): this method involves splicing audio fragments from different sentences to form a new audio file. Application: this is used to produce synthetic speech or create new dialogue scenes, introducing more variability in the audio data. Same splicing (3200 sentences): this technique involves splicing multiple audio clips of the same sentence. Application: this increases the diversity of training data and improves the model’s ability to recognize the same content under varying acoustic conditions. The application of these splicing methods provides diverse training information, thereby enhancing the robustness and generalization capability of the speech model. Ensuring an equal number of data across these methods helps avoid training bias, while a balanced number of statements guarantees sufficient variability of information across different contexts. These auto-editing techniques can significantly benefit areas such as speech synthesis, speech recognition, and other related speech-processing tasks, aiming to improve the performance of models under various scenarios.

Overall, the original audio file contains 3200 sentences; the Chinese and English datasets of CycleGAN voice conversion each contain 6400 sentences, and the automatically spliced Chinese and English datasets each contain 9600 sentences, culminating in a total of 19,200 sentences. These datasets provide a balanced and sound foundation for the subsequent training process. We used four residual blocks in the architecture shown in Figure 2 based on experimental results that demonstrated optimal performance with this configuration. During our experiments, we tested architectures with different numbers of residual blocks. We found that using fewer than four blocks (such as three) led to insufficient model depth, resulting in underfitting and reduced accuracy. On the other hand, increasing the number of blocks beyond four (such as five) did not provide significant performance improvements but did increase computational complexity and training time. Therefore, we selected four residual blocks to achieve the best balance between model efficiency and effectiveness. Additionally, as seen in Table 1, we used not only the LFCC but also the other acoustic features, including Mel-Frequency Cepstral Coefficients (MFCCs) and Constant-Q Cepstral Coefficients (CQCCs). In our experiments, we conducted a comprehensive comparison between different cepstral-related features, including the MFCC, CQCC, and LFCC. These experiments revealed that the LFCC consistently provided a superior performance, especially in detecting tampered audio files. The LFCC was more effective in capturing the subtle, high-frequency distortions introduced by voice tampering techniques such as voice conversion and splicing, particularly in cross-language scenarios. As a result, we decided to focus on the LFCC in the current study.

### 4.2. EER Indicator

The EER [28] is a commonly employed metric in biometric systems and binary classification tasks to evaluate model performance, especially pertinent in verification and authentication scenarios. The EER signifies the point at which the rate of false acceptances (false positives) is equal to the rate of false rejections (false negatives), serving as a crucial indicator of the system’s overall effectiveness.

The EER is determined by locating the point on the Receiver Operating Characteristic (ROC) curve where the false acceptance rate (FAR) and the false rejection rate (FRR) converge. Mathematically, the FAR is defined as FP/FP+TN and the FRR as FN/FN+TP, where FP is the number of false positives, TN is the number of true negatives, FN is the number of false negatives, and TP is the number of true positives. The point where FAR equals FRR is known as the EER, in which EER=FAR=FRR. Within the realm of voice authentication systems, the EER serves as a critical performance metric, evaluating the system’s proficiency in accurately distinguishing between legitimate users and imposters based on voice inputs. A lower EER indicates a more reliable and secure voice authentication system, characterized by reduced instances of unauthorized access and minimized rejections of legitimate users. Therefore, the EER is an indispensable metric for assessing and enhancing the robustness of voice authentication technologies.

## 5. Experimental Results

### 5.1. Performance Comparison Between Three Methods in Different Language

**Figure 4:**
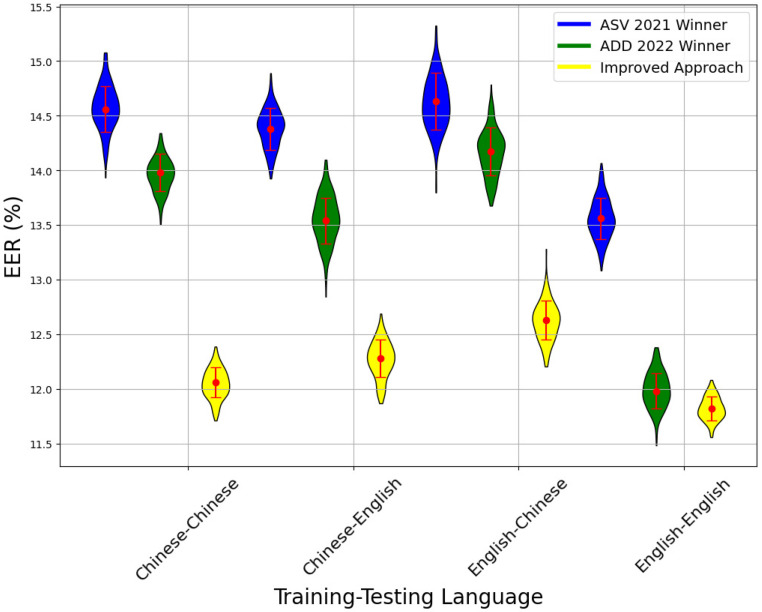
Performance comparison of three methods in different languages.

Figure 4 compares the EER across different language training–testing pairs using three methods: the ASVSpoof 2021 winner, the ADD 2022 winner, and the improved approach. The improved approach consistently outperforms the others, demonstrating notable accuracy and robustness improvements across various linguistic pairings. Specifically, in the Chinese–Chinese and English–English pairs, the improved approach achieves EERs of approximately 12.5% and slightly above 12%, respectively, indicating its effectiveness in both homogeneous and cross-language scenarios. For cross-language pairs such as Chinese–English and English–Chinese, the improved approach continues to excel, highlighting its superior handling of linguistic variations.

**Figure 5:**
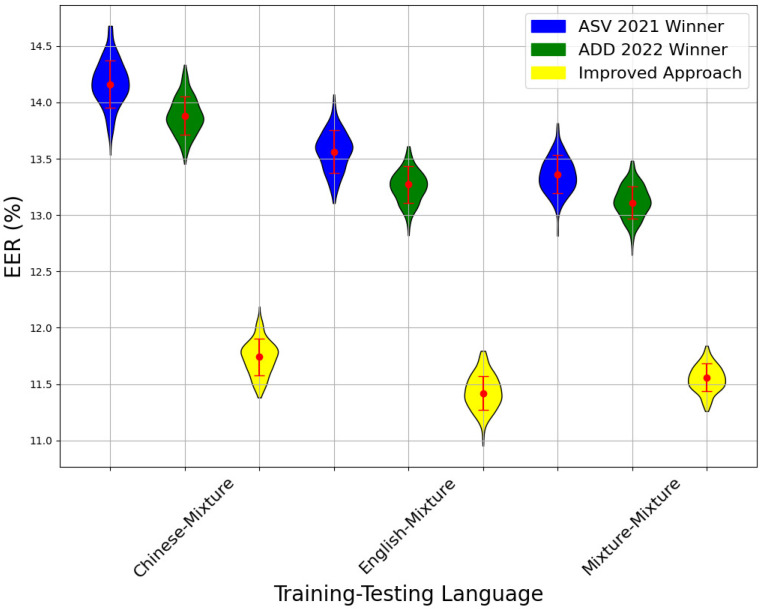
Comparison of performance between three methods in a mixture database.

Furthermore, Figure 5 illustrates the EER comparisons on a mixed database containing both Chinese and English audio files. The improved approach again achieves a lower EER, with significant performance gains in the Chinese–Mixture and English–Mixture pairs, reaching EERs as low as 12% and 11.5%, respectively. Even in the Mixture–Mixture scenario, where both training and testing are conducted on a mixed-language database, the improved approach maintains the lowest EER at around 11.5%.

These results underscore the improved approach’s capability to effectively manage diverse linguistic environments, making it a highly suitable candidate for global voice authentication systems. Its success can be attributed to sophisticated preprocessing, advanced feature extraction, and optimized model architectures, which collectively enhance security and reliability in real-world applications.

In Table 2, we include additional evaluation metrics, such as precision, recall, F1-score, and accuracy, to provide a more comprehensive understanding of the model’s performance. These metrics were calculated for both the validation and testing sets to ensure consistency in detecting tampered audio across different portions of the dataset. As can be seen in Table 2, the model maintains balanced and high performance across precision, recall, and F1-score, while achieving comparable or better results than the competition winners, particularly in cross-language and mixed-language cases. This demonstrates the effectiveness of our architecture in capturing diverse audio patterns, making it more resilient to variations in language and speaker characteristics. This level of stability and adaptability is critical for multilingual tampered audio detection, underscoring our model’s enhanced capability to handle real-world audio verification tasks in a wide range of language environments.

In summary, the improved approach establishes a new benchmark in voice authentication by consistently achieving lower EERs across varied language pairs. This improvement signifies enhanced security and reliability for voice authentication systems operating in dynamic linguistic contexts, underlining the method’s adaptability and effectiveness in a globalized environment.

### 5.2. Cross-Language Robustness Analysis

Table 3 outlines the variations in EER comparisons across single-language and mixed-language test sets for three methods: the ASVSpoof 2021 method, the ADD 2022 method, and the proposed method. The proposed method demonstrates superior performance in all categories, with notable reductions in EER variation. In single-language scenarios, it achieves an EER of 3.08%, compared with 11.72% and 8.46% for the ASV 2021 and ADD 2022 methods, respectively. For mixed-language variations, the proposed method records an EER of 2.16%, significantly lower than the 8.12% and 5.02% from the other methods. Overall, the proposed method maintains the lowest average EER at 2.62%, illustrating its robustness and effectiveness.

These results confirm the efficacy of the proposed method, which leverages advanced preprocessing, sophisticated feature extraction, and optimized model architectures. It sets a new standard in voice authentication, ensuring enhanced security and reliability across diverse linguistic contexts.

### 5.3. Comparison in Voice Conversion Data

**Figure 6:**
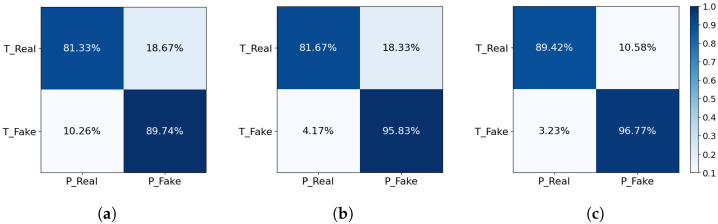
The confusion matrices of baseline method and our improved approach in using voice conversion data. (**a**) ASV 2021 method; (**b**) ADD 2022 method; (**c**) proposed approach.

Figure 6 presents the confusion matrices for the ASVSpoof 2021 method, the ADD 2022 method, and the proposed method, comparing their performance in accurately identifying real and fake audio samples. For the ASVSpoof 2021 method, the confusion matrix reveals that it correctly identifies 81.33% of real audio samples and 89.74% of fake audio samples. However, it misclassifies 18.67% of real samples as fake and 10.26% of fake samples as real.

The ADD 2022 method shows improvement, correctly identifying 81.67% of real audio samples and 95.83% of fake audio samples. The misclassification rates are 18.33% for real samples and 4.17% for fake samples. The proposed method exhibits the best performance among the three. It correctly identifies 89.42% of real audio samples and 96.77% of fake audio samples, with lower misclassification rates of 10.58% for real samples and 3.23% for fake samples.

The results indicate that the proposed method outperforms both the ASVSpoof 2021 and ADD 2022 methods in terms of accuracy. It achieves the highest true positive rates for both real and fake audio samples, and the lowest false positive rates, demonstrating its superior capability to accurately classify audio samples. This performance enhancement can be attributed to advanced preprocessing techniques, sophisticated feature extraction methods, and optimized model architectures employed in the proposed method.

In conclusion, the confusion matrices validate that the proposed method is significantly more effective in distinguishing between real and fake audio samples compared with the baseline methods. This superior performance enhances the reliability and security of the voice authentication system, making it more robust against various types of audio manipulation and spoofing attacks.

### 5.4. Performance Under Different Attack Conditions

**Figure 7:**
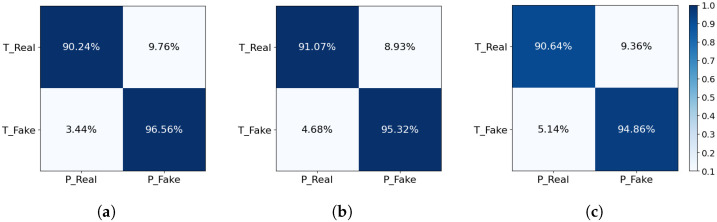
Three methods confusion matrix in different splicing methods. (**a**) Delete. (**b**) Different splicing. (**c**) Same splicing.

Figure 7 presents the confusion matrices for the proposed method applied to three distinct splicing techniques: delete, different splicing, and same splicing. These matrices evaluate the performance of the method in correctly identifying real and fake audio samples.

For the delete method, the confusion matrix indicates that the proposed method correctly identifies 90.24% of real audio samples and 96.56% of fake audio samples. It misclassifies 9.76% of real samples as fake and 3.44% of fake samples as real. For the different splicing method, the proposed method correctly identifies 91.07% of real audio samples and 95.32% of fake audio samples, with misclassification rates of 8.93% for real samples and 4.68% for fake samples. For the same splicing method, the proposed method correctly identifies 90.64% of real audio samples and 94.86% of fake audio samples, with misclassification rates of 9.36% for real samples and 5.14% for fake samples.

The results demonstrate that the proposed method consistently achieves high true positive rates for both real and fake audio samples across all three splicing techniques while maintaining low false positive rates. This superior ability to accurately classify audio samples underlines the method’s consistent performance, highlighting its robustness and effectiveness across different splicing methods.

In conclusion, the confusion matrices validate that the proposed method is highly effective in distinguishing between real and fake audio samples, achieving superior performance across all three splicing scenarios. This enhances the reliability and security of the voice authentication system, making it more robust against various types of audio manipulation and splicing attacks. The success of the proposed method can be attributed to its advanced preprocessing techniques, sophisticated feature extraction methods, and optimized model architectures, all of which contribute significantly to its outstanding performance.

The proposed method consistently outperforms the baseline methods, as demonstrated in Table 4, achieving lower EERs across all tampering techniques. It recorded an EER of 7.11% for delete, 7.76% for different splicing, and 8.03% for same splicing, with an average EER of 7.63%. These results highlight its enhanced capability in accurately detecting and analyzing tampered audio data.

**Figure 8:**
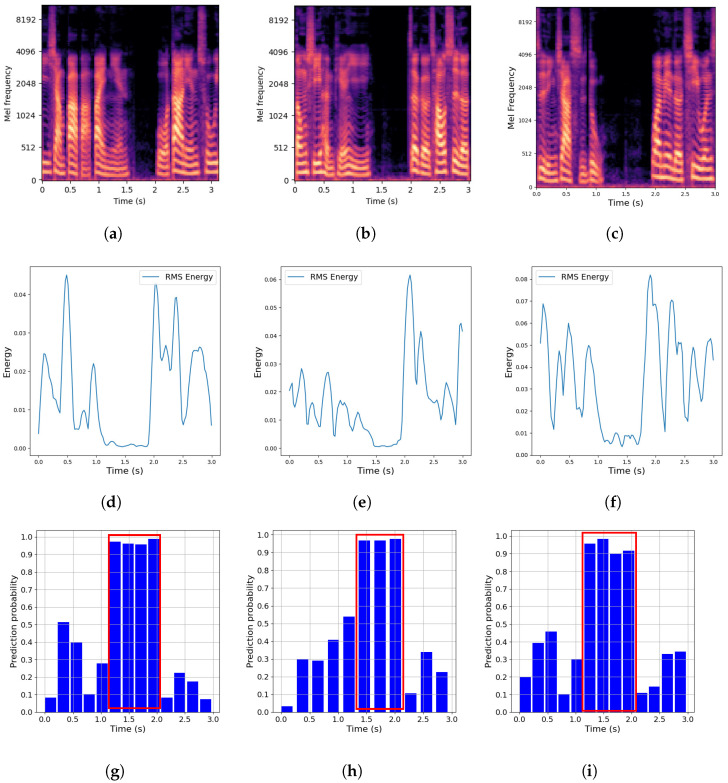
For analysis, one utterance was selected from the delete, different splicing, and same splicing scenarios, yielding nine sub-figures. The top, middle, and bottom rows display the spectrogram, RMS energy, and predicted probability over time, respectively. (**a**) Delete. (**b**) Different splicing. (**c**) Same splicing. (**d**) Delete. (**e**) Different splicing. (**f**) Same splicing. (**g**) Delete. (**h**) Different splicing. (**i**) Same splicing.

Figure 8 presents the spectrogram, Root Mean Square (RMS) energy, and predicted probability, demonstrating the effectiveness of the proposed approach in identifying tampered segments within the input speech. One utterance was chosen from each of the delete, different splicing, and same splicing scenarios for analysis, resulting in a total of nine sub-figures. The RMS energy plot was constructed by calculating the RMS value of each frame and mapping it along the time axis. Identifying tampered parts from the low-level spectrum, as seen in Figure 8a–c, remains challenging, even when translating time frame sequences into energy-based representations (Figure 8d–f). However, the predicted probabilities from the proposed method, displayed in Figure 8g–i, highlight the tampered segments marked by red boxes. As a result, the proposed approach not only determines whether a speech is spoofed but also pinpoints the specific spoofed segments.

## 6. Conclusions

The primary contribution of this paper lies in effectively addressing the challenge of detecting tampered audio within multilingual mixed scenarios, specifically Chinese and English contexts. We proposed an improved approach to cross-lingual and multi-method tampered speech detection by integrating an enhanced ResNet architecture with an LSTM model, which improved the accuracy and generalization capabilities for tampered audio detection, achieving an EER of 11.62%. This performance surpasses that of the models from the ASVSpoof 2021 and ADD 2022 competitions, highlighting the potential of our proposed framework in practical applications. Additionally, we built a bilingual dataset and employed advanced tampering techniques. Despite these advancements, certain limitations remain, particularly in handling more complex multilingual scenarios. Future research will focus on exploring various voice conversion techniques, such as StarGAN-VC and AutoVC, to evaluate their impact on tampered audio detection performance. Additionally, we plan to integrate multimodal features, combining audio and text data, along with incorporating text-to-speech (TTS) technology.

## Figures and Tables

**Table 1:** Comparison of EERs for different acoustic features.

	Metrics	EER
Feature		
MFCC		13.12%
CQCC		12.65%
LFCC		11.62%

**Table 2:** Performance evaluation of the proposed model across Chinese, English, and Mixture language test sets using precision, recall, F1-score, and accuracy.

Test Set	Class	Precision	Recall	F1-Score	Accuracy
Chinese	Real	87.56%	89.78%	88.66%	88.23%
	Fake	89.54%	87.25%	88.38%	
English	Real	89.62%	87.64%	88.62%	88.86%
	Fake	87.63%	89.78%	88.69%	
Mixture	Real	89.65%	87.58%	88.60%	88.54%
	Fake	87.14%	89.63%	88.37%	

**Table 3:** Comparison of variation between three methods in different situations.

Variation	ASV 2021 Winner	ADD 2022 Winner	Proposed Approach
Single language	11.72%	8.46%	**3.08%**
Mixture language	8.12%	5.02%	**2.16%**
Average	9.92%	6.74%	**2.62%**

**Table 4:** EER (%) Performance for three types of tampering methods.

Method	Delete	Different Splicing	Same Splicing	Avg
ASV 2021 Winner	8.66%	8.94%	9.17%	8.92%
ADD 2022 Winner	8.34%	8.51%	8.66%	8.50%
Proposed Approach	**7.11%**	**7.76%**	**8.03%**	**7.63%**

## Data Availability

Data are contained within the article.
